# In Utero Origins of Acute Leukemia in Children

**DOI:** 10.3390/biomedicines12010236

**Published:** 2024-01-19

**Authors:** Adam J. de Smith, Logan G. Spector

**Affiliations:** 1 Center for Genetic Epidemiology, Department of Population and Public Health Sciences, Keck School of Medicine, University of Southern California, Los Angeles, CA 90033, USA; desmith@usc.edu; 2 USC Norris Comprehensive Cancer Center, University of Southern California, Los Angeles, CA 90033, USA; 3 Division of Epidemiology and Clinical Research, Department of Pediatrics, University of Minnesota, Minneapolis, MN 55455, USA

**Keywords:** leukemia, prenatal risk factors, epidemiology, newborn screening, twins

## Abstract

Acute leukemias, mainly consisting of acute lymphoblastic leukemia (ALL) and acute myeloid leukemia (AML), comprise a major diagnostic group among hematologic cancers. Due to the early age at onset of ALL, particularly, it has long been suspected that acute leukemias of childhood may have an in utero origin. This supposition has motivated many investigations seeking direct proof of prenatal leukemogenesis, in particular, twin and “backtracking studies”. The suspected in utero origin has also focused on gestation as a critical window of risk, resulting in a rich literature on prenatal risk factors for pediatric acute leukemias. In this narrative review, we recount the circumstantial and direct evidence for an in utero origin of childhood acute leukemias.

## 1. Descriptive Epidemiology of Childhood Acute Leukemias

**Figure 1:**
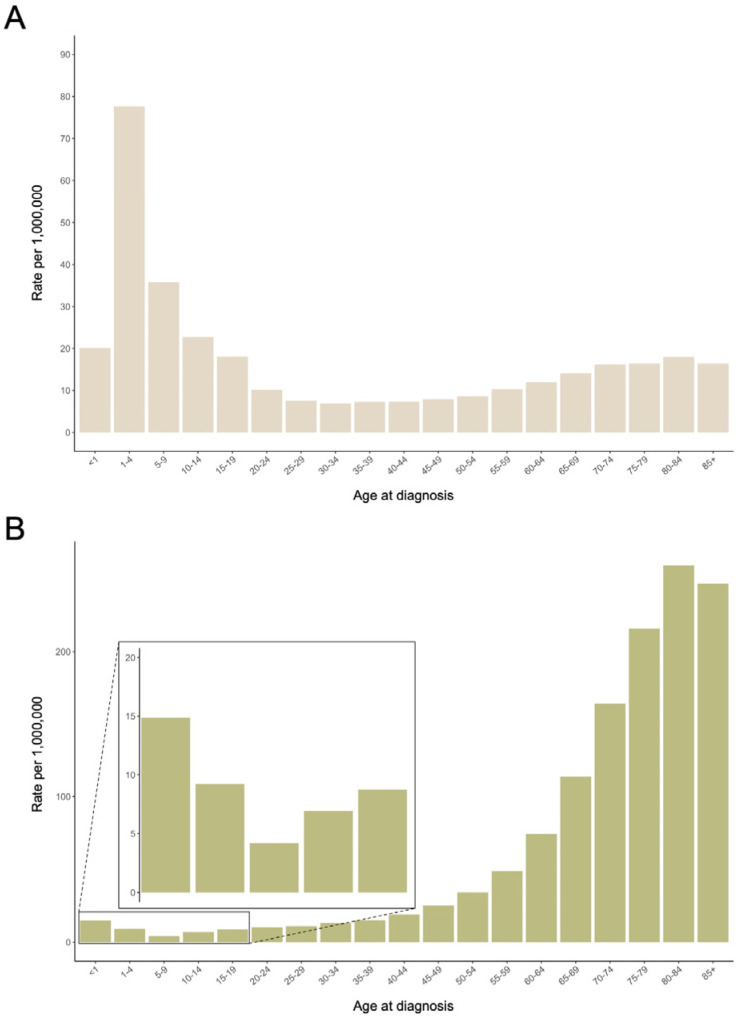
Incidence of acute lymphoblastic leukemia (**A**) and acute myeloid leukemia (**B**), SEER 22, 2000–2020.

In the United States, ALL and AML have distinctive age-related patterns (Figure 1, including adult incidence for comparison) [1]. ALL manifests as a predominantly pediatric disease, with nearly two-thirds of cases occurring in children under the age of 20. Notably, within this age range, a sharp peak incidence is observed between 2 and 5 years of age, while incidence remains lower for the rest of the lifespan. In contrast, AML presents as a disease predominantly affecting adults, with the incidence rising steadily with advancing age and peaking in the elderly population, particularly those above 60 years old.

**Figure 2:**
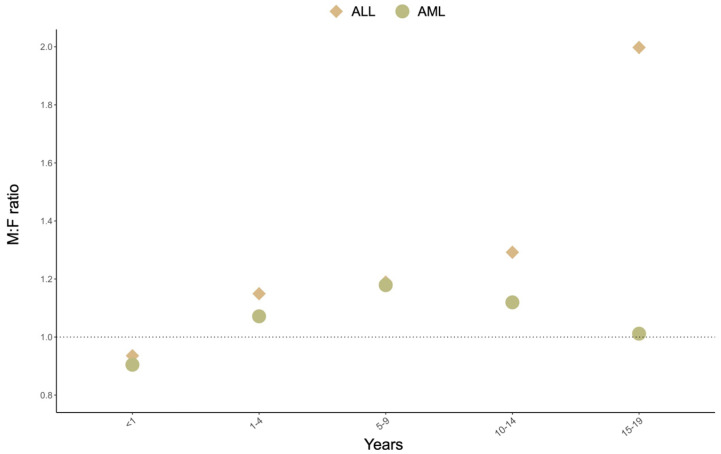
Male-to-female ratio of incidence of childhood acute leukemia, SEER 22, 2000–2020.

Different patterns of incidence by sex also are evident, which are described by the male:female ratio of incidence (M:F) (Figure 2). Both ALL and AML display a slight female preponderance in the first year of life. Thereafter, ALL has a moderately higher rate in males (M:F 1.2–1.4) in early and mid-childhood, while the M:F ratio becomes far larger in late adolescence when the rate among males is double that of females. By contrast, AML incidence shows a more even distribution between males and females, as past infancy and throughout the pediatric period, the M:F ratio is between 1 and 1.2.

There are likewise interesting patterns of incidence by racial and ethnic groups as commonly construed in the United States. In pediatric ALL, the highest rates are seen among Hispanics or Latinos of any race, followed by white and Asian children, and lastly, Black children, whose rates of B-cell ALL, in particular, are half that in other children [2,3]. In the adolescent and young adult age group, the same pattern holds although with even higher rates for Hispanics or Latinos and near parity for white, Black, and Asian individuals. AML shows less variation in incidence by race or ethnicity across the pediatric period. In children, there is a slightly higher rate among Asian–Pacific Islanders compared to other groups [4].

Globally the incidence of acute leukemias mirrors that in the United States. ALL incidence, both within 5-year age groups and age-adjusted, is similar in countries included in the International Agency for Research on Cancer’s Cancer Incidence on Five Continents database [5]. However, IARC has certified several population-based registries in Africa, which confirms a far lower incidence of ALL in sub-Saharan Africa compared to other regions of the world [6]. The incidence of AML also varies across geography but to a lesser extent [7]; the rate in the highest incidence regions is ~2.5 times greater than in the lowest. Males have a greater incidence than females for both ALL and AML in most regions, albeit the magnitude of the M:F ratio varies somewhat.

## 2. Evidence for an In Utero Origin of Acute Leukemias in Brief

Circumstantial evidence for an in utero origin for childhood acute leukemias is threefold. In pediatric acute leukemias, the young age at onset alone, especially for ALL during the early-life peak, suggests prenatal leukemogenesis; since most cancers are thought to require the sequential acquisition of two or more somatic “hits” over time, in children with cancer diagnosed under five years of age, the process is thought to extend into the prenatal period of necessity. However, this presumption of in utero origins diminishes with older age at diagnosis. Acute leukemias occur in developing hematopoietic cells, which are present across the age span, but some populations of cells vulnerable to transformation are known to comprise a much larger proportion of total hematopoietic cells in fetal versus adult life [8]. Lastly, these patterns have focused the epidemiology of childhood on pregnancy as a critical window of risk. The fact that many pathologies of pregnancy, maternal exposures during gestation, and perinatal characteristics have been consistently associated with acute leukemias also circumstantially supports their in utero origin.

There are three lines of direct evidence for an in utero origin for acute leukemias. First, there are a small number (<20) of case reports of prenatal diagnosis of leukemia in the literature [9]. While these reports do not establish the frequency with which acute leukemia develops in utero, they provide definitive proof of the principle that it can happen. Twins, particularly the study of twins concordant for acute leukemia, provide a second line of evidence. Finally, there is a small but robust literature on “backtracking” acute leukemia patients’ somatic alterations to samples taken at birth (i.e., dried blood spots or umbilical cord blood).

Below, we review the evidence from twin studies, backtracking studies, and epidemiologic studies of prenatal risk factors in more depth.

## 3. Twin Studies

The occurrence of concordant childhood leukemia arising in twins was first recorded in the medical literature in 1882 in Germany [10]. By the middle of the 20th century, several case reports of concordant leukemia in twins had been noted, and based on these findings, review papers published in the 1960s and 1970s proposed the prenatal origins of childhood leukemia [10,11,12]. By the beginning of the 21st century, over 70 twin pairs with concordant childhood leukemia were reported in the literature, including at least 37 ALL and 17 AML twin diagnoses (reviewed in Greaves et al., 2003 [10]). The majority of the reported concordant leukemias in twins were diagnosed in children younger than 5 years of age, with approximately half of the concordant pairs being diagnosed with infant leukemia (i.e., <1 year of age), a proportion of total childhood leukemias much higher than that found in singleton cases.

The concordance rate for acute leukemia in monozygotic (MZ) twins, reported to be between 5% and 25%, is vastly higher than the concordance rate of leukemia in dizygotic, or fraternal, twins, in whom concordant leukemias are exceedingly rare. Such a large difference was unlikely to be due to a shared genetic predisposition in MZ twins, and instead, a proposed mechanism that could plausibly explain the high concordance rate of leukemia in MZ twins was their shared placental vasculature [10,12,13]. Approximately 60% of MZ twins develop in a single monochorionic placenta, which leads to vascular anastomoses that facilitate the passage of blood cells between the circulation of each twin [14]. Thus, it was proposed that an initiating leukemogenic lesion could develop in one twin and then be transferred to the other through the shared placental vasculature [12].

This hypothesized mechanism of twin-to-twin transfusion of preleukemic clones was subsequently confirmed by molecular genetic analyses of acute leukemias in concordant twin pairs. In particular, the detection of identical fusion events in MZ twins concordant for leukemia supports a common clonal origin, given that the independent generation of leukemia-forming translocations with identical breakpoints in both twins is highly unlikely. Ford and colleagues first demonstrated this in three pairs of MZ twins concordant for *KMT2A*-rearranged infant ALL, whereby each twin pair shared the same-sized restriction fragments of *KMT2A* [15]. Subsequently, several additional MZ twin pairs concordant for ALL were identified with identical gene fusion events, including for *ETV6-RUNX1* [16,17,18], *BCR-ABL1* [19], and *TCF3-ZNF384* translocations [20]. In addition, MZ twins concordant for high hyperdiploid ALL, which is the most common molecular subtype of ALL and is characterized by the gain of particular chromosomes, have also been identified. In these twin pairs, the sharing of unique IGH and TCR sequences in their leukemia cells supports the prenatal development of high hyperdiploid ALL in one of the twins and subsequent transfer to the other twin [21,22]. Furthermore, the in utero origins of infant AML were also confirmed in MZ twins concordant for *KMT2A* rearrangements [23].

Studies of leukemia in twins have also been informative regarding the timing of mutational events that initiate or drive the progression to overt leukemia in children. In twins concordant for infant leukemia, the latency period between the diagnosis of leukemia in both twins is relatively short. However, a wide variation in age of leukemia diagnosis has been observed in twins concordant for childhood ALL, with latency periods of up to 14 years reported in one twin pair [10,17]. This supports a two-hit mechanism of leukemogenesis, whereby the initiating lesion develops prenatally, and then secondary somatic mutations arise in the postnatal period, resulting in progression to acute leukemia. Genetic studies in twins concordant for ALL have provided strong evidence for this two-hit model, with several reported instances of distinct secondary somatic mutations and copy-number alterations in twins that share the same gene fusion or other initiating events [22,24,25]. Further support is provided by twins discordant for childhood ALL, in which preleukemic clones harboring the initiating lesion, such as *ETV6-RUNX1* fusion, have been found to persist for several years in the healthy co-twin without leading to the development of leukemia, supporting the supposition that additional mutations are required for overt leukemia development [19,24,26,27]. The timing of mutational events that drive the development of childhood AML is less clear, as fewer twin pairs with AML have been investigated, although a protracted latency period between the onset of AML in at least one concordant twin pair has been reported [28].

Although the focus of this review is on the prenatal origins of childhood acute leukemias, it is important to note that hematological malignancies in adult twin pairs also suggest the prenatal origins of some adult-onset leukemias. For example, in 1929, a pair of identical twin brothers were both diagnosed with chronic lymphocytic leukemia (CLL) at the age of 56 years [29]. More recently, a pair of MZ twins were diagnosed with primary myelofibrosis, a type of myeloproliferative neoplasm (MPN), at the ages of 37 and 38 years and were found to share the same somatic mutation in the *CALR* gene [30]. Lineage tracing of the MPN clone via whole-genome sequencing in each twin revealed a shared clonal origin, supporting the prenatal origins and transplacental transmission of this mutation. This remarkable finding in relation to an adult-onset chronic hematologic malignancy suggests that some acute leukemias in adults may also have their origins in utero, and further studies of MZ twins concordant for adult acute leukemias are also warranted.

## 4. Backtracking Studies

**Figure 3:**
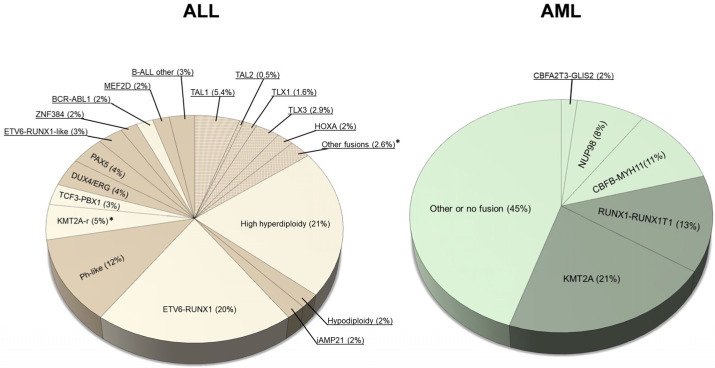
Molecular subtypes of childhood leukemia and their backtracking status. Frequencies of ALL subtypes (**left**) were modified from Pui et al., 2019 [31]. We highlight ALL subtypes that have been previously backtracked in light beige, with remaining B-cell and T-cell ALL subtypes that have not been backtracked colored in darker beige and T-cell subtypes highlighted by cross-hatching. Some T-cell ALL patients with *KMT2A*-rearranged (*KMT2A*-r) and other mutation events have been backtracked, indicated by asterisks. Frequencies of AML subtypes (**right**) were modified from Huang et al., 2022 [32], with previously backtracked subtypes highlighted in olive green and non-backtracked subtypes colored light green.

Since twins with acute leukemia are rare, whether concordant or discordant, further knowledge of the in utero origins of childhood leukemia has been gained through investigating the presence of leukemia-forming somatic alterations in blood samples obtained at birth in children who later went on to develop the disease. Such “backtracking” studies were made possible due to the widespread availability of newborn dried bloodspots (DBSs, or “Guthrie cards”), collected by health services from newborn heel pricks for the original purpose of screening for neonatal health conditions, in addition to the development of polymerase chain reaction (PCR) methods that could detect somatic alterations in DNA at low copy numbers. Figure 3 summarizes the frequency of primary somatic mutations in ALL and AML [31,32] and indicates which have been backtracked. We expand upon this history below.

Gale et al. (1997) first described the detection of *KMT2A-AFF1* (formerly, *MLL-AF4*) translocations in newborn DBSs obtained from three patients who were diagnosed with ALL under the age of two years, thus confirming the prenatal development of this initiating lesion among these very young patients [33]. The backtracking of a *KMT2A-MLLT10* translocation in the newborn DBS of a two-year-old childhood AML patient was later described [34]. In a seminal study by Wiemels et al. (1999), patient-specific PCR primers for the *ETV6-RUNX1* translocation were used for backtracking in the newborn DBS samples in childhood ALL patients known to harbor this fusion gene at diagnosis [35]. Of nine patients tested, with age-at-diagnoses ranging from 2 to 5 years, six had detectable *ETV6-RUNX1* fusion sequences in their DBS, thus corroborating the findings in twins that this genetic lesion may often develop prenatally. In another backtracking study by Wiemels and colleagues, they investigated the prenatal origins of the *RUNX1-RUNX1T1* (formerly, *AML1-ETO*) translocation in childhood AML and detected the fusion gene in newborn DBS in five out of ten patients; two of these positive patients were over the age of 10 years, providing support for a two-hit model of leukemogenesis in some AML patients [36].

Following the use of PCR primers to amplify fusion events in backtracking studies of patients with translocations such as *ETV6-RUNX1*, other methods were required to investigate the prenatal origins of chromosomal aneuploidies that drive some childhood leukemias. High hyperdiploidy, which is the most common subtype of childhood ALL and is characterized by a nonrandom gain of chromosomes, has a similar peak age-at-diagnosis as *ETV6-RUNX1* at around 2 to 5 years of age, which suggests possible in utero origins. Several studies have used patient-specific PCR primers designed to target leukemia clonotypic immunoglobulin heavy chain (IgH) or T-cell receptor (TCR) sequences, confirming the prenatal development of high hyperdiploidy [37,38,39]. Indeed, in the largest series of childhood ALL patients backtracked to date, preleukemic clones were detectable in newborn DBSs in 10 out of 11 high hyperdiploid ALL patients [39,40]. The prenatal origins for other ALL subtypes either remain inconclusive, as is the case for *TCF3-PBX1* [41,42], or have yet to be backtracked, such as *BCR-ABL1*-like (Ph-like) ALL [43,44] and other relatively recently described molecular subtypes including rearrangements of *DUX4* [45,46], *ZNF384* [47], and *MEF2D* [48]. Similarly, the in utero origins of most childhood AML-defining rearrangements have yet to be investigated.

**Figure 4:**
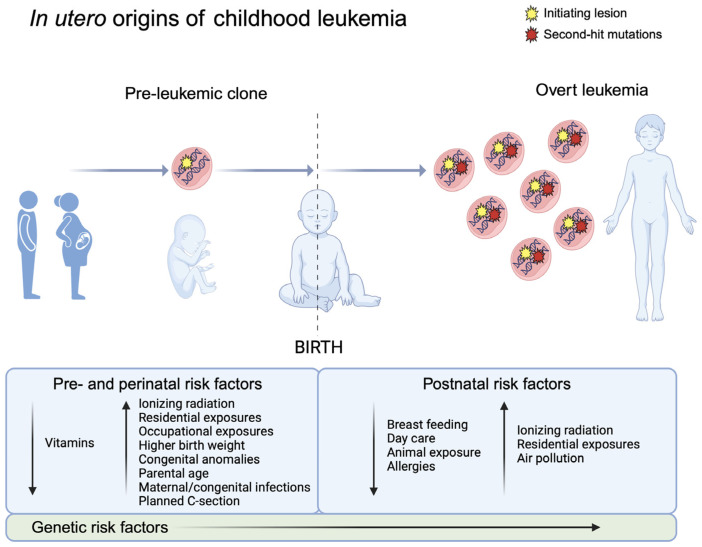
Natural history of childhood leukemia development supports in utero origins. Evidence from monozygotic twins concordant for childhood leukemia and the backtracking of somatic alterations to newborn blood samples support the prenatal development of initiating lesions in childhood leukemia (**left**), e.g., *ETV6-RUNX1* fusions in childhood ALL. In the two-hit model of childhood leukemia development, the postnatal acquisition of secondary somatic mutations is followed by clonal expansion, leading to the development of overt acute leukemia (**right**). We summarize reported prenatal and postnatal risk factors for childhood leukemia, which provide potential opportunities for disease prevention. Prenatal risk factors may contribute both to the development of the initiating lesion in utero as well as the later progression to leukemia, whereas the effects of postnatal factors are likely limited to the risk of acquiring secondary mutations and clonal expansion. Postnatal protective factors such as daycare and exposure to animals may represent proxies of infectious exposure and are supportive of Greaves’ “delayed infection” hypothesis of childhood leukemia development [26]. We note that this list of pre- and postnatal risk factors is not comprehensive; the list is derived from Lupo and Spector, 2020 [60]. Germline genetic risk factors seem to play a role in both the development of pre-leukemic clones and the progression to overt leukemia. Created with Biorender.com.

As well as confirming the prenatal origins of several childhood leukemia subtypes, backtracking studies have also provided evidence supporting the two-hit model of leukemogenesis, with second-hit somatic mutations and gene deletions found to arise postnatally [19,49,50,51] (Figure 4). Further evidence for both the in utero origins of childhood leukemia and the postnatal development of second-hit mutations has been provided by the screening of leukemia-initiating events in healthy newborns. This has been the subject of recent reviews [52,53], but in brief, the *ETV6-RUNX1* and *TCF3-PBX1* fusions have been detected in DBSs in up to 5% and 0.6% of healthy newborns, respectively, frequencies much higher than the incidences of these leukemia subtypes, thus supporting that additional mutations are required for progression to overt leukemia.

We note that myeloid leukemia in children with Down syndrome (ML-DS), classified as an independent category of AML by the World Health Organization, has a unique natural history that demonstrates both in utero origins and the two-hit model of leukemogenesis [54]. ML-DS typically develops by the age of 4 years [55] and is always preceded by the prenatal development of somatic mutations in the hematopoietic transcription factor gene *GATA1* that result in transient leukemia with or without clinical manifestations (transient abnormal myelopoiesis, TAM, or “silent TAM”, respectively) [56]. These pathognomonic *GATA1* mutations are detectable in all patients with ML-DS, but postnatally acquired secondary mutations are necessary to drive the transformation of transient leukemia to ML-DS [57,58]. Trisomy of chromosome 21 clearly predisposes the development of *GATA1* mutations in utero [54], but little is known regarding risk factors for the development of ML-DS in a subset of individuals with transient leukemia.

## 5. Prenatal Risk Factors for Acute Leukemia in Childhood

Twin and backtracking studies have clearly focused epidemiologic investigations of childhood acute leukemia on pregnancy (Figure 4). The prenatal period appears to be susceptible to translocations as well as chromosomal aneuploidies that can lead to childhood leukemia—whether this is because of the vulnerabilities of specific cell types in early hematopoiesis or due to particular exposures during pregnancy that may drive the formation of these genetic lesions remains to be determined. The role of developmental hematopoiesis in the prenatal origins of childhood leukemia has been reviewed elsewhere [8,59]. Here, we review select, major associations of prenatal risk factors, grouped roughly as pathologies of pregnancy, maternal exposures, and perinatal characteristics, with childhood acute leukemias. Paternal pre-conceptional exposures, which are presumed to work by causing mutations in sperm, are not covered since they do not exert their effect during gestation. In this section, we concentrate on reports of meta-analyses or large, pooled analyses, which summarize a body of work rather than individual studies and thus offer more definitive conclusions about association and potential causality. Furthermore, we focus on prenatal and perinatal risk factors of childhood leukemia because of the topic of this review, but we also summarize some of the reported postnatal risk factors [60] in Figure 4 for context.

### 5.1. Pathologies of Pregnancy

The association of childhood leukemia with maternal diabetes, both pre-existing and gestational, as well as with the correlated trait of body mass index, was the subject of a recent systematic review and meta-analysis by Marley et al. [61]. Through the aggregation of the results of 34 studies, the authors found that any maternal diabetes during pregnancy raised the risk of ALL (odds ratio (OR) = 1.46, 95% confidence interval (CI) = 1.28 to 1.67), even after including only analyses adjusted for birth weight (OR = 1.74, 95% CI = 1.29 to 2.34). Maternal pre-pregnancy body mass index was positively associated with any leukemia in childhood (OR per five-unit increase in body max index = 1.07, 95% CI = 1.04 to 1.11). There were no associations of maternal diabetes or body mass index with AML, specifically.

Maternal infection in pregnancy and the risk of leukemia in offspring has been the subject of both a recent systematic review and meta-analysis [62] and a pooled analysis of six birth cohorts [63]. The former aggregated results of 20 manuscripts published prior to 2018, which reported on maternal infection during pregnancy or congenital infection of infants (which would have to have been acquired during pregnancy). Most studies reported a positive association (OR > 1) for an infection-related variable, but few reported an “any infection” variable, thus precluding a global assessment. However, in smaller subsets of studies that examined particular infections, there were significant associations of influenza during pregnancy with ALL (OR = 3.64, 95% CI = 1.34 to 9.90) and childhood leukemia (OR = 1.77, 95% CI = 1.01 to 3.11) as well as varicella (OR = 10.19, 95% CI = 1.98 to 52.39) and rubella (OR = 2.79, 95% CI = 1.16 to 6.71) infections with childhood leukemia. In contrast to the mostly retrospective, case–control studies included in the meta-analysis, the International Childhood Cancer Cohort Consortium pooled data from six prospective studies to examine maternal infection and childhood leukemia risk. Maternal urinary tract infection was significantly associated with any childhood leukemia (hazard ratio (HR) = 1.68, 95% CI = 1.10 to 2.58) and suggestively but non-significantly with ALL (HR = 1.49, 95% CI = 0.87 to 2.56) and AML (HR = 2.70, 95% CI = 0.93 to 7.86). Maternal respiratory infection was significantly associated with any childhood leukemia (HR = 1.57, 95% CI = 1.06 to 2.34) and AML (HR = 2.37, 95% CI = 1.10 to 5.12), while the ALL association was merely suggestive (HR = 1.43, 95% CI = 0.94 to 2.19).

### 5.2. Maternal Exposures

Diagnostic irradiation in pregnant women has long been suspected of contributing to leukemia risk in their offspring. Wakeford and Bithell reviewed studies conducted over 65 years [64]. A meta-analysis of case–control and case–cohort studies found a significantly higher risk of any childhood leukemia among the offspring of mothers who were exposed to diagnostic irradiation during pregnancy (relative risk (RR) = 1.28, 95% CI = 1.16 to 1.41) and in particular those who were exposed to diagnostic irradiation to the abdomen (RR = 1.82, 95% CI = 0.90 to 3.66). These associations are generally taken to be causal, resulting in successful efforts to reduce the use of diagnostic irradiation with pregnant women [65].

The Childhood Cancer and Leukemia International Consortium (CLIC) has conducted several large, pooled analyses of maternal occupational and residential exposures during pregnancy. Maternal occupational exposure to pesticides was not associated with ALL (OR = 1.01, 95% CI = 0.78 to 1.30) but was strongly and significantly associated with AML (OR = 1.94, 95% CI = 1.19 to 3.18) [66]. Neither ALL nor AML was significantly associated with maternal occupational paint exposure during pregnancy [67]. Home pesticide exposure during pregnancy was significantly associated with both childhood ALL (OR = 1.43, 95% CI = 1.32 to 1.54) and AML (OR = 1.55, 95% CI = 1.21 to 1.99) [68]. Home paint exposure during pregnancy was significantly associated with ALL, albeit to a lesser degree than pesticides (OR = 1.14, 95% CI = 1.04 to 1.25).

CLIC also investigated maternal prenatal vitamin use in a large, pooled analysis [69]. Maternal vitamin use during pregnancy was associated with a reduced risk of childhood ALL (OR = 0.85, 95% CI = 0.78 to 0.92) as was folic acid use (OR = 0.80, 95% CI = 0.71 to 0.89). There was a suggestion of an inverse association of AML with maternal vitamin (OR 0.92, 95% CI: 0.75 to 1.14) and folic acid use (OR = 0.68, 95% CI: 0.48 to 0.96) but neither was significant. These associations did not seem to vary by trimester of use.

Although there are persistent questions about the validity of retrospective recall of diet, including during pregnancy [70], the association between maternal diet and childhood acute leukemia has been the subject of a recent meta-analysis [71]. There was a significant, inverse association between maternal fruit consumption and ALL (OR = 0.71, 95% CI: 0.59 to 0.86) and a suggestion of an inverse association with maternal vegetable consumption (OR = 0.88, 95% CI: 0.69 to 1.11); the number of studies with data for AML was insufficient to evaluate the association. Associations of ALL with maternal grain, dairy, and processed meat intake were null, while associations with AML were not evaluable. Enough studies examined maternal coffee and tea consumption during pregnancy to allow meta-analysis. There was a significant, positive association of ALL with maternal coffee consumption (OR = 1.45, 95% CI: 1.12 to 1.89) but not tea consumption (OR = 0.95, 95% CI: 0.79 to 1.14); both associations were null for AML.

Perhaps surprisingly, maternal tobacco use is not associated with either ALL or AML in meta-analyses [72]. By contrast, maternal alcohol use is notably associated with AML in the most recent comprehensive meta-analysis [73], as the authors note that there was, “…a statistically significant dose-response association of any level of maternal alcohol consumption compared with nondrinking during pregnancy exclusively with acute myeloid leukemia (AML) (ORmoderate consumption = 1.64, 95% CI: 1.23–2.17 and ORhigh consumption = 2.36, 95% CI: 1.60–3.49)”. Dose–response associations are considered one hallmark of causality [74]. However, there was again no association with ALL.

### 5.3. Perinatal Characteristics

Perinatal characteristics, meaning those measured around the time of birth, are often interpreted as indicators of the health of the pregnancy (e.g., birth weight) and are therefore included in this review.

The association of birth weight with childhood acute leukemia has been apparent for decades [75]. The latest meta-analysis of the topic included 28 studies published through 2021 [76]. As has long been known, high birth weight (>4000 g) was associated with a higher risk of ALL (OR = 1.28, 95% CI: 1.20 to 1.35) compared to normal birth weight (2500–4000 g), while low birth weight (<2500 g) was associated with lower risk (OR = 0.83, 95% CI: 0.75 to 0.92). There was a null association of high and low birth weight with AML, although the association with high birth weight was suggestive (OR = 1.23, 95% CI: 0.97–1.56). Others have refined this analysis to examine size for gestational age [77] or percent of optimal birth weight [78], but the overall findings that larger babies have a somewhat higher risk of ALL remains the case. Interestingly, the degree of association between birth weight and ALL seems to differ by molecularly defined subtype [79].

Higher parental age at birth of offspring is consistently associated with both ALL and AML. CLIC has produced the most comprehensive analyses of parental age and ALL [80] and AML [81] to date. Focusing on the population-based studies created by record linkage, which would not suffer from selection bias, there was a significant association of maternal age with ALL (OR per 5-year increase = 1.05, 95% CI: 1.01–1.08). Similarly, there was an increased risk of AML among children of mothers > 40 years of age at delivery (OR = 6.87, 95% CI: 2.12–22.25). Neither study identified associations with paternal age, although since maternal and paternal age are highly correlated, it is difficult to disentangle them. Paternal age and, to a lesser extent, maternal age are both associated with a higher burden of de novo mutations in offspring [82], which is a plausible but as-yet unexamined [83] explanation for the epidemiologic association of childhood acute leukemia with parental age. There are, however, many demographic, obstetric, and behavioral correlates of maternal age [84] which raise the question of the extent to which confounding affects maternal age associations with childhood acute leukemias.

Structural birth defects are typically detected at or near birth but clearly originate during gestation. CLIC examined the association between childhood acute leukemia and structural birth defects in a large, pooled analysis of over 15,000 cases and 46,000 controls [85]. Interesting findings of association included digestive system defects and ALL (OR = 2.70, 95% CI: 1.46–4.98), congenital heart defects and AML (OR = 2.86, 95% CI: 1.81–4.52), and nervous system defects and AML (OR = 4.23, 95% CI: 1.50–11.89). Notably, the authors found a higher prevalence of birth defects in registry-based studies than in questionnaire-based ones, and effect sizes were generally larger in registry-based studies. Another large, pooled analysis of record-linkage studies in the United States found significant, moderately strong (OR 1.01–5.0) associations of ALL with integument and musculoskeletal defects and of AML with musculoskeletal, genitourinary, digestive, respiratory, and circulatory defects [86]; although children with chromosomal anomalies associated with both birth defects and acute leukemia, particularly Down syndrome, were excluded from the analysis, there is still the possibility these findings were partially due to undetected such anomalies.

Lastly, the mode of birth has been extensively studied. Birth by cesarean section can be an indicator of the health of a pregnancy but also is an event with many consequences of its own, most obviously the seeding of newborns with a very different microbiome than in vaginal birth [87,88]. CLIC conducted the largest and most nuanced pooled analysis of the association of mode of birth with acute leukemia [89]. Contrary to expectations, given the microbiome differences between the two modes of birth, birth by cesarean section was not associated with either ALL (OR = 1.06, 95% CI: 0.99–1.13) or AML (OR = 0.99, 95% CI: 0.84–1.17). However, when the timing of surgery was considered, a significant association of pre-labor cesarean section with ALL emerged (OR = 1.23, 95% CI: 1.04–1.47). While it remains possible that newborn microbiotic exposures may differ between planned and emergency cesarean sections, an alternative hypothesis was also born. Labor involves the release of maternal and infant cortisol, which is lower in cesarean sections and lower still in pre-labor cesarean sections [90]. Glucocorticoids are analogs of cortisol and a backbone of ALL therapy [91]. One intriguing explanation for these results is thus that exposure to cortisol during labor may cull pre-leukemic cells, which share a vulnerability to this class of molecules with overt leukemia.

## 6. Opportunities for Prevention

Natural history studies of childhood acute leukemias in twins and backtracking studies have clearly revealed that most disease has an initiating somatic event in utero and a secondary event postnatally. Consequently, a fair number of maternal exposures during gestation and pregnancy characteristics appear to modify the risk of acute leukemia in offspring. The aggregate data are sufficient to suggest several potential routes for screening and prevention of childhood acute leukemia.

We have recounted several modifiable prenatal risk factors for childhood acute leukemia. Promoting behaviors or exposures that confer lower risk would thus be a form of primary prevention. There also could be efforts to create prenatal risk indices based on the known risk factors for offspring leukemia, modifiable or not. These could be enhanced with the addition of maternal genetics that influence offspring risk of leukemia, an almost entirely unexamined area of research. To date, there has been very little epidemiology of pre-leukemia, owing in large part to the cumbersome assays for detecting it. The development of more robust, scalable assays for pre-leukemia at birth—in DBSs since they are the most abundant newborn sample—would enable widespread studies evaluating the association of prenatal risk indices with the detection of pre-leukemia.

Screening for pre-leukemia at birth seems potentially feasible from a technical standpoint but will face ethical and practical challenges. Most notably, any test is likely to be probabilistic rather than deterministic, and many leukemias occur past infancy; both these facts would seem to disqualify acute leukemia from inclusion in newborn screening programs, although norms are changing. Presumably, newborns with detectable pre-leukemia at birth would be at higher risk of developing overt leukemia, but this will need to be confirmed by prospective natural history studies, which have yet to be conducted.

If newborn screening for childhood acute leukemia were enabled, the end goal would be prevention. One could envision attempting early detection by periodic sampling of blood to detect any change in translocation prevalence. However, this faces hurdles with compliance since sampling blood from babies and small children is generally avoided. More importantly, it may not be possible to sample often enough to capture the clonal expansion of fully transformed acute leukemia cells, which is generally thought to occur in a matter of days to a few weeks. Consequently, it is considerably more feasible to target children at higher risk of acute leukemias with efforts to either reduce their prevalence of pre-leukemia or reduce their likelihood of acquiring a secondary mutation that would tip them into progression to overt leukemia. Postnatal risk factors are coming into focus [60], many of which are modifiable and may suggest interventions. For instance, CLIC has shown breastfeeding to be protective for both ALL and AML [92], thus it may be particularly fruitful to promote breastfeeding among children at the highest risk of developing leukemia. Others have suggested applying preventative efforts more widely, such as microbiome modification to help prime the developing immune system and prevent overactive immune responses to childhood infections [26,93,94,95]. Lastly, research to identify currently unknown vulnerabilities in pre-leukemic cells could result in novel preventative strategies not yet imagined.

## 7. Conclusions

Studies of leukemia in twins and the backtracking of somatic alterations in patient newborn blood samples have confirmed the in utero origins of childhood leukemia, across several subtypes of both ALL and AML. The identification of potentially modifiable risk factors in the prenatal period presents a window of opportunity for the primary prevention of childhood acute leukemia. Additional work is needed to determine the timing of the development of childhood leukemia subtypes that have not yet been examined, which will inform the extent to which we may be able to screen for and potentially prevent all childhood leukemias.
